# Incidence of postpartum hemorrhage based on the improved combined method in evaluating blood loss: A retrospective cohort study

**DOI:** 10.1371/journal.pone.0289271

**Published:** 2023-07-28

**Authors:** Fangyuan Zheng, Haiyan Wen, Lan Shi, Caihe Wen, Qiumeng Wang, Shouzhen Yao

**Affiliations:** 1 Department of Obstetrics, Hangzhou Women’s Hospital, Hangzhou, China; 2 Department of Fourth Clinical Medical College, Zhejiang Chinese Medical University, Hangzhou, China; MUHAS: Muhimbili University of Health and Allied Sciences, UNITED REPUBLIC OF TANZANIA

## Abstract

**Objective:**

In view of the current clinical inaccuracies and underestimations of postpartum hemorrhage amount, this study aims to investigate the incidence, etiology, clinical characteristics of postpartum hemorrhage in different modes of delivery based on the combination of volumetric method, gravimetric method and area method in evaluating blood loss.

**Design:**

This retrospective cohort study was conducted in Hangzhou Women’s Hospital from January 2020 to June 2021, including 725 cases of postpartum hemorrhage among 18,977 parturients. Based on different modes of delivery, the participants were divided into three groups: vaginal delivery, forceps delivery, and cesarean section, for comparison.

**Methods:**

Using an improved combined assessment method for blood loss, we retrospectively analyzed a cohort of parturients with postpartum hemorrhage who underwent vaginal delivery, forceps delivery, or cesarean section and were hospitalized in Hangzhou Women’s Hospital from January 2020 to June 2021.

**Results:**

(1) Among the 18,977 parturients, 725 cases of postpartum hemorrhage occurred, with an incidence rate of 3.8%, and severe postpartum hemorrhage accounted for 0.4% of the cases. (2) The incidence of postpartum hemorrhage was significantly higher in the forceps delivery group than in the vaginal delivery group (χ^2^ = 19.27, *P*<0.001), while the incidence of severe postpartum hemorrhage was significantly higher in the cesarean section group than in the vaginal delivery group (χ^2^ = 8.71, *P* = 0.003). (3) The causes of postpartum hemorrhage were statistically different among the different delivery modes, with varying underlying factors (*P*<0.001). (4) Patients with postpartum hemorrhage in different delivery modes showed statistically significant differences in age, body mass index (BMI), birth weight, gestational age, gravidity, parity, the decline of postpartum peripheral blood hemoglobin concentration, and estimated blood loss (*P*<0.05). (5) The proportion of blood transfusion was significantly higher in the cesarean section group than in the vaginal delivery and forceps delivery groups (χ^2^ = 231.03, *P*<0.001).

**Limitations:**

This study is a single-center retrospective study, which may have led to selection bias in case selection. Additionally, the implementation of the combined three blood loss assessment methods may not have been strictly followed in all cases. Moreover, due to the mixing of bleeding with amniotic and irrigation fluids, the accuracy of evaluation may have been affected, leading to the possibility of inaccuracy of blood loss.

**Conclusions:**

Forceps delivery and cesarean section increase the risk of postpartum hemorrhage, but forceps delivery does not significantly increase the incidence of severe postpartum hemorrhage. Uterine atony remains the leading cause of postpartum hemorrhage, while birth canal laceration and placental factors are the second most common causes of postpartum hemorrhage in forceps delivery and cesarean section, respectively. In this study, the volumetric method, gravimetric method and area method were combined to quantitatively assess postpartum hemorrhage amount. The combined method has strong clinical practicability and is less affected by subjective factors, although it also has limitations. In the future, we still need to focus on the early prediction and identification of postpartum hemorrhage, and further improve the quantitative assessment of postpartum blood loss.

## Introduction

Postpartum hemorrhage (PPH) is a serious obstetric complication that occurs and develops so rapidly, which is still a leading cause of maternal death in China [1]. It can be accompanied by serious maternal complications, such as hypovolemic shock, disseminated intravascular coagulation (DIC), acute renal failure, acute respiratory distress, Sheehan syndrome; loss of fertility has also been reported [2, 3]. In the past 10 years, the incidence of PPH in some developed countries was still rising, mainly due to the increased incidence of uterine atony [4]. It is also positively correlated with labor induction, prenatal oxytocin use, and the increasing cesarean section rate [5]. A meta-analysis involved multiple countries in 2012 indicated that the incidence of PPH fluctuated from 7.2% to 25.7% (with an average of approximately 10.8%).The study also pointed out that the incidence of PPH was affected by the regions and the methods of blood loss assessment [6]. At present, clinical underestimation of postpartum blood loss is still common [7–10], and some studies indicated that inaccurate estimates of actual blood loss after birth occurred by healthcare providers are the main reason for the delayed bleeding response [11–13]. The traditional visual method to estimate blood loss during childbirth and postpartum is subjective and not accurate enough [14–16]. Quantitative measurements of blood loss such as spectrophotometry [17, 18], gravimetric measurement [9], objective quantification with a novel birthing drape using the volumetric method [8, 19], hematocrit [20, 21] are more accurate than the visual method. However, no method of quantifying blood loss has been proven to be optimal by the existing data [22]. Therefore, how to improve the accuracy of postpartum blood loss assessment still needs to be further explored.

After the opening of the second-child policy in China, the number of pregnant women with advanced age, scarred uterus re-pregnancy, and pernicious placenta previa increases significantly. The composition of the childbearing population has changed, which also changes the causes of PPH [23]. The maternal mortality rate caused by PPH in China is significantly higher than that in developed countries [24, 25]. At present, there is still a big gap in the ability to prevent and treat PPH. Based on the improved combined assessment method of blood loss, this study retrospectively analyzed the clinical data of 725 patients with PPH in our hospital, and aimed to explore the incidence, etiology, clinical characteristics in different delivery modes.

## Methods

### 1. Study design and participants

**Fig 1:**
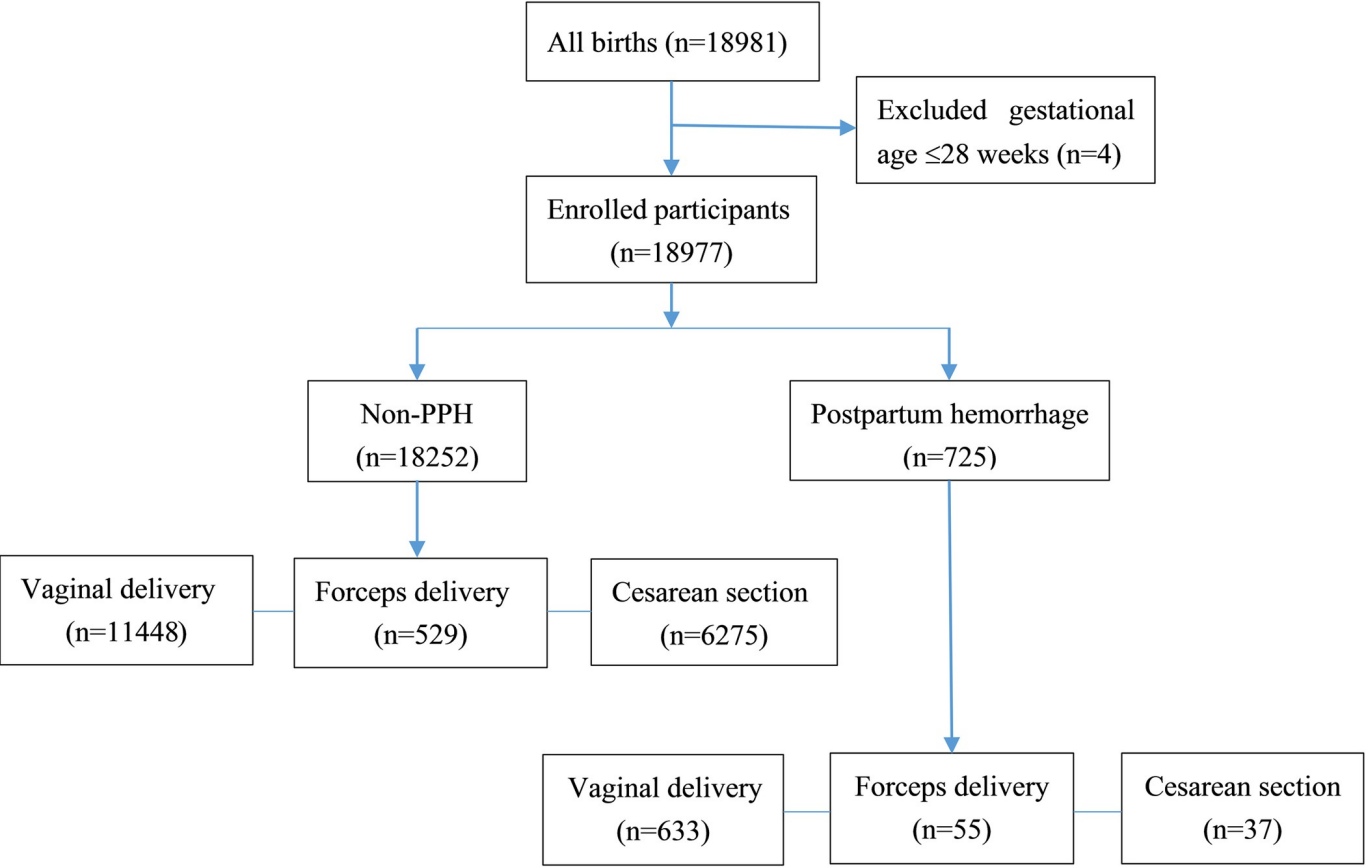
Flow diagram of the total study population, exclusions and subdivisions.

This retrospective study was conducted in Hangzhou (population more than 10,000,000), the capital of Zhejiang Province, which is among one of the developed areas in southeast coast of China with high medical level. The annual number of births from 2018 to 2020 is above 13,000 in Hangzhou Women’s Hospital. The people served by our hospital are mainly from cities with high education level, and most of them have college degree or above. The study cohort consisted of vaginal delivery, forceps delivery, and cesarean section parturients who were hospitalized in Hangzhou Women’s Hospital between January 2020 and June 2021 and met the inclusion criteria. The inclusion criteria required participants to have a gestational week of delivery of ≥28 weeks and to have given birth in the hospital. Participants with severe liver and kidney dysfunction, severe hematological diseases (excluding anemia and simple thrombocytopenia), or incomplete information were excluded. Blood loss, incidence, and causes of postpartum hemorrhage in different delivery groups (vaginal delivery, forceps delivery, and cesarean section groups) were compared using an improved combined assessment method of blood loss, which is a standardized method for estimating blood loss during delivery that includes both visual and quantitative assessments. Fig 1 provides detailed information about the study population.

### 2. Ethics approval and consent to participate

This study was conducted in accordance with International Code of Ethics for Biomedical Research Involving Human and Helsinki Declaration, and the study protocol was approved by the Ethical Review Committee of Hangzhou Women’s Hospital in China, approval number [2021K10-01]. Because of the retrospective nature of the study, patient consent for inclusion was waived. The identity of the participants are kept anonymous, and the information in this study is kept strictly confidential.

### 3. Clinical data collection

From January 2020 to June 2021, all the clinical data were collected from electronic medical records system used in our hospital, including the age, gravidity, parity, body mass index (BMI), gestational age at delivery, birth weight, placental abnormalities (including placental adhesion, placenta accreta, placenta previa, retained placenta and placental abruption), pregnancy complications, hemoglobin level concentration within one week before delivery and peripheral blood hemoglobin concentration 24 to 48 hours postpartum, indications for labor induction, forceps and cesarean section, estimate blood loss, causes of PPH blood transfusion (type and amount of blood transfusion) and intervention measures (type and dose of uterotonics, uterine tamponade, vascular ligation, hysterectomy). The general data of patients with postpartum hemorrhage as blood loss, incidence and causes of postpartum hemorrhage in different delivery methods (vaginal delivery group, forceps delivery group, cesarean section group) were compared.

### 4. Definition of postpartum hemorrhage

PPH is defined as blood loss ≥500 mLs following vaginal birth or ≥1000 mLs following caesarean section within 24 hours. Severe PPH refers to blood loss ≥1000 mLs or hypovolemic shock within 24 hours after delivery [26].

### 5. Quantitative assessment methods of postpartum blood loss

We adopted a unified blood loss assessment method and conducted regular pre-job training. We made detailed records of the intra-partum and 24-hour postpartum bleeding of each delivery. There are complementary advantages among volumetric method, gravimetric method and area method. The combined method can be divided into the following three steps. Firstly, the volume of the blood collection basin used in vaginal delivery and the suction bottle used in cesarean section can be directly read (the volume of amniotic fluid and irrigating fluid need to be deducted). During vaginal delivery, when the amniotic fluid had basically flowed completely after fetal birth, then the blood collection basin was placed under the maternal buttocks to collect blood loss. The medical covering drape used in cesarean section is waterproof, which can collect amniotic fluid and blood loss effectively. During cesarean section, the assistant used a negative pressure aspirator to collect as much amniotic fluid as possible after the rupture of amniotic membrane and record the amount of amniotic fluid in the suction bottle. Then removed the amount of amniotic fluid when calculating the blood loss in the suction bottle. Secondly, the remaining bleeding on the operating table was calculated by the area method (10cm×10cm is referred as 10 mL). At last, the perineal pad was placed under maternal buttocks until 24 hours after delivery. The perineal pad would be replaced several times, and the final weight was calculated totally (blood volume (mL) = (the weight of the pad used—the weight before use)/1.05). The sum of the blood loss in the above three steps is the total blood loss within 24 hours after delivery.

### 6. Statistical analysis

Statistical analyses were performed using IBM SPSS Statistics 19.0 (IBM Corp., Armonk, NY, USA). The Kolmogorov-Smirnov test was used for the normality test. The data conforming to the normal distribution are represented by the mean ± standard deviation. The data of the skewed distribution are represented by the quartile, and categorical variables in numbers and percentages (%). Independent sample analysis of differences between groups was performed using variance analysis, χ^2^ test, Fisher’s exact test or nonparametric test (Mann Whitney U test and Kruskal Wallis test). Multiple comparisons were performed using Bonferroni method or Dunnett T3 method. The level of significance was set at a probability value of *P* < 0.05.

## Results

### 1. Participant characteristics

The total population consisted of 18981 deliveries who were hospitalized in our hospital from January 2020 to June 2021. 18977 cases were enrolled after four were excluded for gestational age less than 28 weeks. PPH occurred in 725 cases (76 cases with severe PPH), and all of them were cured and discharged. The total incidence of PPH was 3.8%, while that of severe PPH was 0.4%. The estimated blood loss was at least 500 mLs and at most 5500 mLs. The age of patients was 20 to 43 years old, with an average age of 29.64±3.57 years. There were 505 cases of primipara and 220 cases of multipara. There included 718 cases of singleton pregnancy and 7 cases of twin pregnancy. Among them, PPH occurred in 633 of 12081 cases in vaginal delivery and 55 of 584 cases in forceps delivery. And there were 37 cases with severe PPH occurred in 6312 cases of cesarean section. The indications for cesarean section were as follows: 14 cases of scarred uterus, 9 cases of placental factors (including placenta previa, placenta accreta, and placental abruption), 5 cases of trial labor failure, 4 cases of twin pregnancy, 1 case of intrauterine infection, 1 case of severe preeclampsia, 1 case of macrosomia, 1 case of breech, and 1 case of elderly primipara.

### 2. Comparison of the incidence of postpartum hemorrhage in different delivery methods

In vaginal delivery group and forceps delivery group, the incidence of PPH was 5.2% (633/12081) and 9.4% (55/584) respectively, while the incidence of severe PPH was 0.3% (36/12081) and 0.5% (3/584) respectively. The incidence of severe PPH in the cesarean section group was 0.6% (37/6312). The incidence of PPH in the forceps delivery group was significantly higher than that of vaginal delivery group (χ^2^ = 19.27, *P*<0.001). There were statistical differences in the incidence of severe PPH in different delivery methods (χ^2^ = 8.83, *P* = 0.012) (Table 1). The incidence of severe PPH in the cesarean section group was significantly higher than that of vaginal delivery group (χ^2^ = 8.71, *P* = 0.003). But there was no significant difference in the incidence of severe PPH between forceps delivery group and vaginal delivery group, and between forceps delivery group and cesarean section group (*P*>0.05).

**Table 1:** Comparison of the incidence of severe postpartum hemorrhage in different delivery methods.

	Total	Cases of severe PPH	Incidence of severe PPH (%)	χ^2^ test	
				χ^2^	*P*
vaginal delivery	12081	36	0.3	8.83	0.012
forceps delivery	584	3	0.5		
cesarean section	6312	37	0.6^a^		

a: Compared with vaginal delivery group, χ^2^ = 8.71, *P* = 0.003

### 3. The causes of postpartum hemorrhage in different delivery methods

The common causes of PPH can be classified into four aspects: uterine atony, placental factors, birth canal lacerations and coagulation defects. The causes of PPH in this study were as follows: uterine atony accounted for 84% (609/725), birth canal lacerations accounted for 10.2% (74/725), placental factors accounted for 5.5% (40/725) and coagulation defects accounted for 0.3% (2/725). The percentage of causes of PPH was statistically different in different modes of delivery (*P*<0.001) (Table 2). The primary reason of PPH in the vaginal delivery group was uterine atony (accounted for 87.2%), which was significantly different from the forceps delivery group and the cesarean section group (χ^2^ = 25.83, *P*<0.001; χ^2^ = 18.01, *P*<0.001). The main causes of PPH in forceps delivery group were uterine atony (accounted for 61.8%) and birth canal lacerations (accounted for 34.5%). The proportion of birth canal laceration was higher than that in vaginal delivery group and cesarean section group (χ^2^ = 57.85, *P*<0.001; χ^2^ = 16.08, *P*<0.001). The main causes of PPH in cesarean section group were uterine atony (accounted for 62.2%) and placental factors (accounted for 35.1%). The proportion of placental factors was higher than that in vaginal delivery group and forceps delivery group (χ^2^ = 35.25, *P*<0.001; χ^2^ = 16.11, *P*<0.001).

**Table 2:** The causes of postpartum hemorrhage in different delivery methods.

The causes of PPH	Delivery method [n (%)]			χ^2^ or Fisher’s exact test	
	Vaginal delivery	Forceps delivery	Cesarean section	Statistic	*P*
Uterine atony	552 (87.2)^a^	34 (61.8)	23 (62.2)	38.10	<0.001
Placental factors	25 (3.9)	2 (3.6)	13 (35.1)^b^	34.66	<0.001
Birth canal lacerations	55 (8.7)	19 (34.5)^c^	0	30.91	<0.001
Coagulation defects	1 (0.2)	0	1 (2.7)	5.466	0.105
Total	633 (100%)	55 (100%)	37 (100%)	-	

a: Compared with forceps delivery group and cesarean section group, χ^2^ = 25.83 *P*<0.001; χ^2^ = 18.01, *P*<0.001; b: Compared with vaginal delivery group and forceps delivery group, χ^2^ = 57.85, *P*<0.001;χ^2^ = 16.08, *P*<0.001; c:Compared with vaginal delivery group and cesarean section group, χ^2^ = 35.25, *P*<0.001;χ^2^ = 16.11, *P*<0.001.

### 4. Comparison of clinical data and estimated blood loss of patients with postpartum hemorrhage in different delivery modes

In this study, the age, body mass (BMI), birth weight, gestational age, gravidity, parity, peripheral blood hemoglobin concentration drop, and estimated blood loss of patients with PPH in different delivery modes were statistically different (*P*<0.05) (Table 3). Furthermore, the age, gravidity, parity and estimated blood loss of patients with PPH in the cesarean section group were higher than those of the vaginal delivery group (*P*<0.01) and the forceps group (*P*<0.01). However, there was no significant difference in estimated blood loss of patients with PPH between the vaginal delivery group and the forceps delivery group (*P* = 0.078). The proportion of blood transfusion in cesarean section is significantly higher than that in vaginal delivery and forceps delivery (χ^2^ = 231.03, *P*<0.001).

**Table 3:** Comparison of clinical data, blood loss and blood transfusion of patients with postpartum hemorrhage in different delivery modes.

	Vaginal delivery	Forceps delivery	Cesarean section	ANOVA	Pearson χ^2^	Kruskal Wallis	*P*
				F value		H value	
Age	29.55±3.46	28.71±3.35	32.62±4.25	15.59	-	-	<0.001
BMI (kg/m^2^)	26.43±3.14	25.05±3.29	26.33±2.61	4.89	-	-	0.008
Birth weight (g)	3450 (3230, 3700)	3470 (3240, 3700)	3200 (2675, 3600)	-	-	6.09	0.048
Gestational age (day)	276 (273, 280)	280 (273, 281)	266 (254, 273)	-	-	41.33	<0.001
Gravidity	2 (1, 2)	1 (1, 2)	2 (2, 3.5)	-	-	35.15	<0.001
Parity	1 (1, 2)	1(1, 1)	2 (1, 2)	-	-	30.31	<0.001
Hb drop (g/L)	28.24±12.18	31.12±9.08	32.19±13.10	3.68	-	-	0.03
Estimated blood loss (ml)	700 (635, 785.5)	656 (610, 825)	1300 (1122.5, 1539)	-	-	94.99	<0.001
Blood transfusion (%)	2.8 (18/633)	5.5 (3/55)	64.9 (24/37)	-	231.03	-	<0.001

The values of Age, BMI and Hb drop are represented by the mean ± standard deviation. The values of Birth weight, Gestational age, Pregnancy times, Parity and Estimated blood loss are represented by the quartile.

## Discussion

### 1. The quantitative assessment of postpartum blood loss

At present, there is no absolutely accurate method to assess postpartum blood loss. Both the gravimetric method [9, 27] and the volumetric method (a surgical drape marked with scale lines placed under maternal buttocks) [8, 19] are more accurate than the visual method. A combined method (direct measurements of spilled blood and sucker bottle volumes, and weighing of surgical towels and drapes before and after use) is considered feasible to evaluate the blood loss during cesarean section [28], but it takes 35~45 minutes. In this study, we adopted an improved quantitative assessment method of postpartum blood loss combining volumetric method, gravimetric method and area method. This improved assessment method integrates the advantages of the three assessment methods, which is less affected by the subjective factors of the evaluator. Compared with the traditional visual method, the accuracy of blood loss assessment is further improved, and the operation is simple and no cumbersome. The easy processing procedures and the least experimental equipment ensure the timeliness of the assessment of postpartum blood loss, so it is believed that the clinical practicality is strong. However, this method also has some limitations. The bleeding is easily mixed with amniotic fluid and irrigation fluid which interferes with the accuracy of evaluation. The measurement is started after the neonatal delivery and the amniotic fluid collected completely before placenta delivery can reduce measurement errors. During vaginal delivery, when the amniotic fluid had basically flowed completely, then the blood collection basin was placed under the maternal buttocks to collect blood loss. When the placenta was delivered, only a small amount of amniotic fluid would flow into the basin, which had little impact on the evaluation of postpartum blood loss. During cesarean section, the assistant used a negative pressure aspirator to collect as much amniotic fluid as possible after the rupture of amniotic membrane and record the amount of amniotic fluid in the suction bottle. Then removed the amount of amniotic fluid when calculating the postpartum blood loss in the suction bottle. When a patient is suffering massive bleeding within a short period of time, it is difficult to collect all the blood loss, which will also increase the measurement error of the blood loss. Other commonly used methods for evaluating postpartum hemorrhage include shock index (SI), laboratory tests (peripheral blood hemoglobin concentration and hematocrit indirectly predict blood loss). A multicenter retrospective study on "Shock Index and Postpartum Hemorrhage in Vaginal Delivery" pointed out that sensitivity of SI is low, and clinical judgment must include other vital signs and symptoms related to hypovolemic shock [29]. Le Bas Abigail et al. suggested that the normal SI range should be 0.7–0.9. SI greater than 1.0 seems to be a useful aid for estimating blood loss in severe PPH and predicting the demand for blood transfusion [30]. Peripheral blood hemoglobin concentration and hematocrit are easily affected by many factors, such as blood concentrating in the early stage of PPH, haemodilution following massive intravenous infusions during PPH and blood transfusion. Thus, the changes in hemoglobin concentration and hematocrit are often not accurately reflected the actual amount of bleeding [31]. So the significance of laboratory testing lies more in the monitoring the patient’s condition and evaluating the effectiveness of treatments, rather than the diagnosis of acute PPH. At present, it is difficult to accurately estimate the amount of postpartum blood loss, and the underestimation of the amount of blood loss may delay the rescue opportunity. Therefore, the early prediction and recognition of postpartum hemorrhage is more important.

At present, there are several studies focusing on the construction of early prediction models for the risk of PPH [32–36]. These models include risk factors such as advanced age, macrosomia, multiple fetus, multiple parturition, length of labor, delivery methods, scarred uterus, placental implants, and hypertensive disorder complicating pregnancy, pregnancy with uterine fibroids, anemia and other independent risk factors for PPH. These risk assessment tools screen out patients at high risk for PPH, which will help improve preparedness for PPH, strengthen monitoring patient and early identification of PPH, and the use of preventive measures to reduce the occurrence of PPH.

### 2. The effects of different delivery modes on the incidence of postpartum hemorrhage

The overall incidence of PPH in this study was 3.8%, and the incidence of severe PPH was 0.4%, which is much lower than previous literature reported (varies from 7.2% to 25.7%) [6].The incidence of postpartum hemorrhage is related to the regions, local medical level, the attention to prenatal examinations and the awareness of prevention of postpartum hemorrhage. Our hospital is a specialized hospital of gynecology and obstetrics in Hangzhou, representing the leading medical level in China. Our hospital attaches great importance to postpartum hemorrhage and takes a variety of measures to reduce the occurrence of postpartum hemorrhage, including weight management during pregnancy to prevent the occurrence of macrosomia, screening high-risk factors of postpartum hemorrhage, the use of uterotonic in the third stage of labor, refined management of the labor process, improvement of the evaluation method of postpartum blood loss and the treatment drill of postpartum hemorrhage.

Analysis of the incidence of PPH in different delivery methods showed that the incidence of PPH in forceps delivery was significantly higher than that of vaginal delivery (9.4% vs 5.2%, *P*<0.001), but it did not significantly increase the incidence of severe PPH (0.5% vs 0.3%, *P*>0.05), and the incidence of severe PPH is between vaginal delivery and cesarean section. Compared with vaginal delivery, the incidence of severe PPH in the cesarean section group was significantly higher (0.6% vs 0.3%, *P* = 0.003). This difference is not only associated with the cesarean section procedure but also with the pregnancy complications experienced by the patients who undergo cesarean section delivery. Most pregnant women have pregnancy complications (such as severe preeclampsia, complete placenta previa, placenta accreta, etc.), advanced age, multiple pregnancies, history of cesarean section and other high-risk factors for PPH. At the same time, studies have confirmed that the risk of PPH of emergency cesarean section is further increased than that of elective cesarean section [37, 38].

### 3. The analysis of causes of postpartum hemorrhage

The common causes of PPH can be classified into four aspects: uterine atony, placental factors, birth canal lacerations and coagulation dysfunction. The four major causes can coexist or be causal to each other. This study showed that PPH caused by uterine atony, birth canal laceration, placental factors and abnormal blood coagulation accounted for 84%, 10.2%, 5.5%, and 0.3% respectively. Uterine atony is still the main cause of PPH, but there are differences in the causes of PPH in different way of delivery. Uterine atony is the primary reason in vaginal delivery group. Uterine atony and birth canal lacerations are the major reasons in forceps delivery group. In the cesarean section group, uterine atony and placental factors are the major factors. The observed difference may be attributed to an increased risk of birth canal lacerations during forceps delivery, as well as a tendency among high-risk patients with placental factors (such as placenta previa and placenta accreta) to opt for cesarean delivery. Uterine atony is still the prime cause of PPH in China. Therefore, powerful uterotonics are the first choice for the treatment of PPH [39]. In recent years, the decrease in maternal mortality caused by PPH is mainly attributed to the increase in the proportion of blood transfusion and perinatal hysterectomy [4, 40]. Obstetric hysterectomy can be adopted when other measures for severe postpartum hemorrhage are ineffective (such as uterine packing, B-Lynch suture, etc.) or cannot be obtained. There is currently no standards and evidence-based basis on how much postpartum blood loss requires hysterectomy [2]. In this study, a patient with estimated blood loss of 5500 mL was diagnosed with amniotic fluid embolism, and finally underwent hysterectomy after medication, massive blood transfusion, and uterine packing.

### Limitations

As a single-center retrospective study, this study may have selection bias in case selection. Moreover, it is challenging to ensure that the three combined blood loss assessment methods are strictly implemented in all cases. Additionally, bleeding can be mixed with amniotic fluid and irrigation fluid, which can interfere with the accuracy of evaluation. As a result, there is a possibility of inaccuracy the blood loss in this study. And in terms of data analysis, there is a lack of adjustment analysis for demographic factors.

## Conclusions

In summary, this study confirmed that forceps delivery and cesarean section increased the risk of PPH, while forceps delivery did not significantly increase the incidence of severe PPH. Uterine atony remains the leading cause of PPH, while birth canal laceration and placental factors are the second most common causes of PPH in forceps delivery and cesarean section, respectively. In this study, the combination of volumetric method, gravimetric method and area method was used to improve the accuracy of quantitatively assessment of postpartum blood loss. In practice, it is found that this method shows strong clinical practicability and is not affected by subjective factors, although it still has limitations. In the future, we still need to continue to focus on the early prediction and identification of PPH, and further modify the quantitative assessment of postpartum blood loss.

## Supporting information

S1 Data (XLSX)
